# Supplementary material for: Pulse irradiation synthesis of metal chalcogenides on flexible substrates for enhanced photothermoelectric performance
Source: Nat Commun. 2024 Jan 25;15:728. doi: 10.1038/s41467-024-44970-4 (PMC10810900; doi:10.1038/s41467-024-44970-4)
Supplement: Supplementary file 1 — Supplementary Information [file 41467_2024_44970_MOESM1_ESM.pdf]

Supplementary Information

Pulse irradiation synthesis of metal chalcogenides on flexible substrates for enhanced photothermoelectric performance

Yuxuan Zhang[1], You Meng[2,*], Liqiang Wang[3], Changyong Lan[4], Quan Quan[1], Wei Wang[1], Zhengxun Lai[1], Weijun Wang[1], Yezhan Li[1], Di Yin[1], Dengji Li[1], Pengshan Xie[1], Dong Chen[1], Zhe Yang[5], SenPo Yip[6], Yang Lu[7], Chun-Yuen Wong[5,*], Johnny C. Ho[1,2,6,*]

[1] *Department of Materials Science and Engineering, City University of Hong Kong, Hong Kong SAR 999077, P.R. China.*
[2] *State Key Laboratory of Terahertz and Millimeter Waves, City University of Hong Kong, Hong Kong SAR 999077, P.R. China.*
[3] *Department of Mechanical Engineering, City University of Hong Kong, Hong Kong SAR 999077, P.R. China.*
[4] *State Key Laboratory of Electronic Thin Films and Integrated Devices, University of Electronic Science and Technology of China, Chengdu 610054, P. R. China.*
[5] *Department of Chemistry, City University of Hong Kong, Hong Kong SAR 999077, P.R. China.*
[6] *Institute for Materials Chemistry and Engineering, Kyushu University, Fukuoka 816 8580, Japan*
[7] *Department of Mechanical Engineering, The University of Hong Kong, Hong Kong SAR 999077, P.R. China.*
[*] *Corresponding author: Johnny C. Ho (johnnyho@cityu.edu.hk); Chun-Yuen Wong (acywong@cityu.edu.hk); You Meng (youmeng2@cityu.edu.hk)*

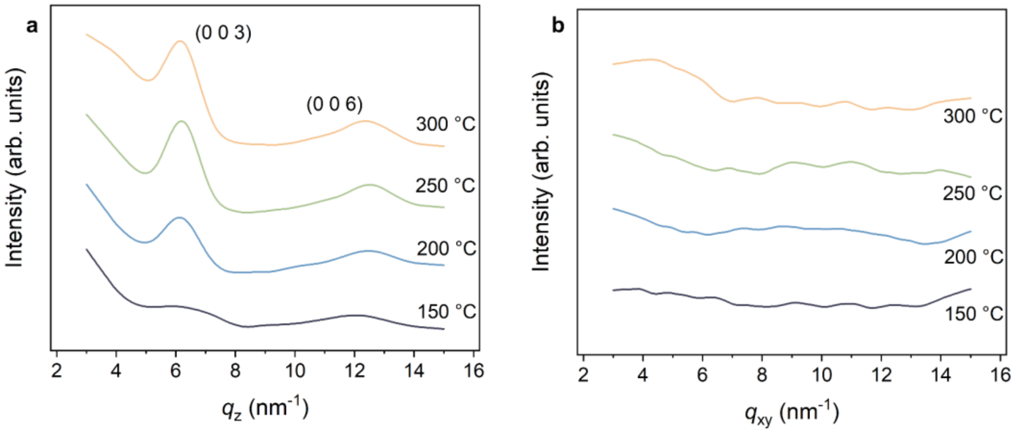

Supplementary Figure 1 | In-plane and out-of-plane diffraction profiles. The intensity profiles corresponding to the GIWAXS patterns in Fig. 2a-d along the q_z direction (out-of-plane, **a**), and q_{xy} direction (in-plane, **b**).

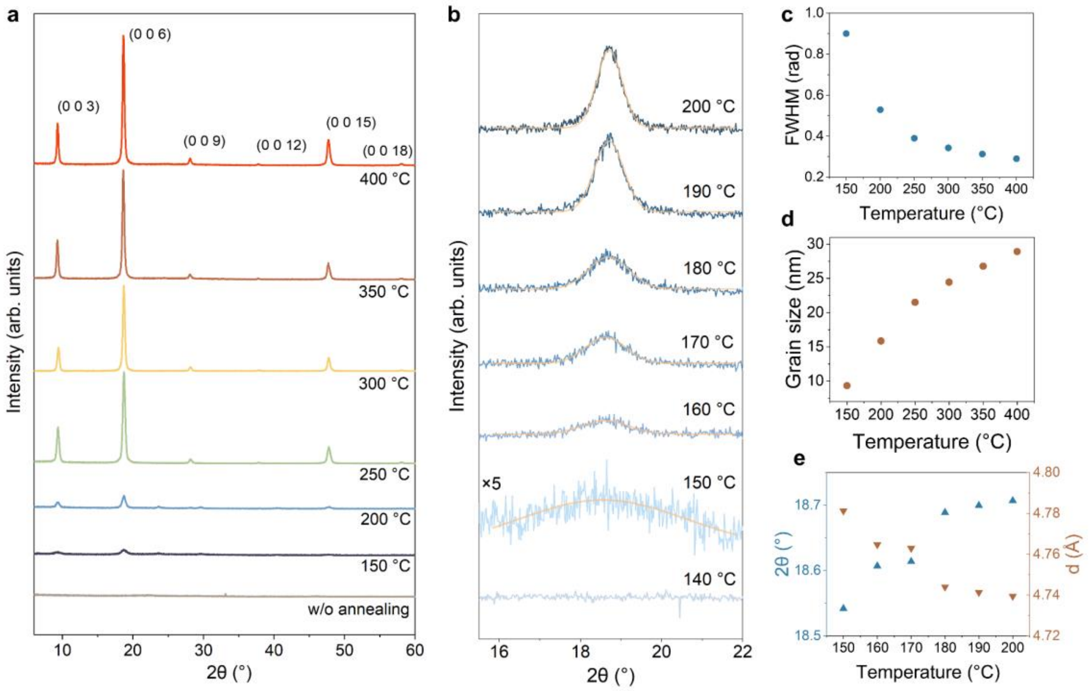

Supplementary Figure 2 | XRD patterns and corresponding evolutions for grain growth. a, XRD spectra of Bi_2Se_3 with different PIS temperatures. **b**, Detailed profile of the (0 0 6) peak with smaller PIS temperature steps (from 140 to 200 °C). **c,d**, Extracted full width at half maximum (FWHM) (**c**) and grain size (**d**) based on (**a**). **e**, 2θ position and interplanar spacing of (0 0 6) plane extracted from (**b**).

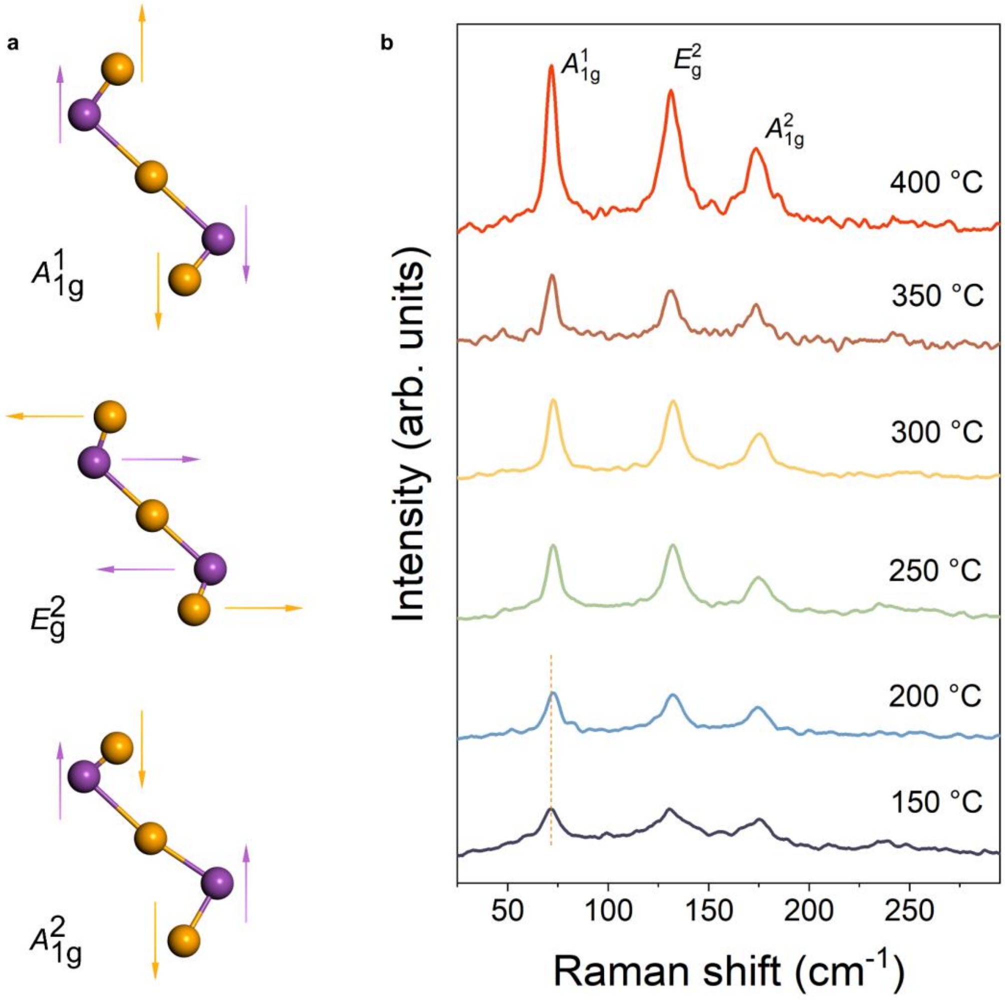

Supplementary Figure 3 | Raman spectra of films via PIS synthesis. a, Vibrational normal modes of the quintuple Bi_2Se_3 layer. **b**, Raman spectra of the Bi_2Se_3 film with different PIS temperatures.

Supplementary Note 1

Layered Bi_2Se_3 has a rhombohedral crystal structure belonging to the space group $R\bar{3}m$. Most reported Raman-active modes are two out-of-plane A_{1g}^1 and A_{1g}^2 vibrations and one in-plane E_g^2 vibration, which are distinguishable at 71.5, 174.4, and 131.7 cm^{-1}, respectively[1]. Corresponding atomic displacements of each vibration are shown in Supplementary Fig. 3a. Three vibration modes intensify with higher annealing temperatures, while A_{1g}^1 and E_g^2 modes develop a slight blue shift from 150 to 200 °C. The pronounced broadening of vibration modes is partly explained by the phonon confinement, where the location of the phonon becomes more certain as the size of nanoparticles (NPs) becomes smaller, and therefore the energy of the phonon must be less certain[2]. This phenomenon is also consistent with the uncertainty rule. The shift of vibration modes is commensurate with the corresponding stress or strain induced in the material. With the growth of NPs, stacked atomic layers enhance the interlayer van der Waals interactions that influence the effective restoring forces acting on QLs, which results in the compression between QLs along the z-axis, which directly explains the blue shift of A_{1g}^1 modes[3]. Concurrently, the phonon hardening due to layer-to-layer stacking also affects the intralayer bonding for the in-plane E_g^2 modes. This effect enhances the electron-phonon coupling with more QLs and causes the blue shift[4]. These results are well consistent with the 1D profile of GIWAXS (Supplementary Fig. 1) and XRD spectra (Supplementary Fig. 2).

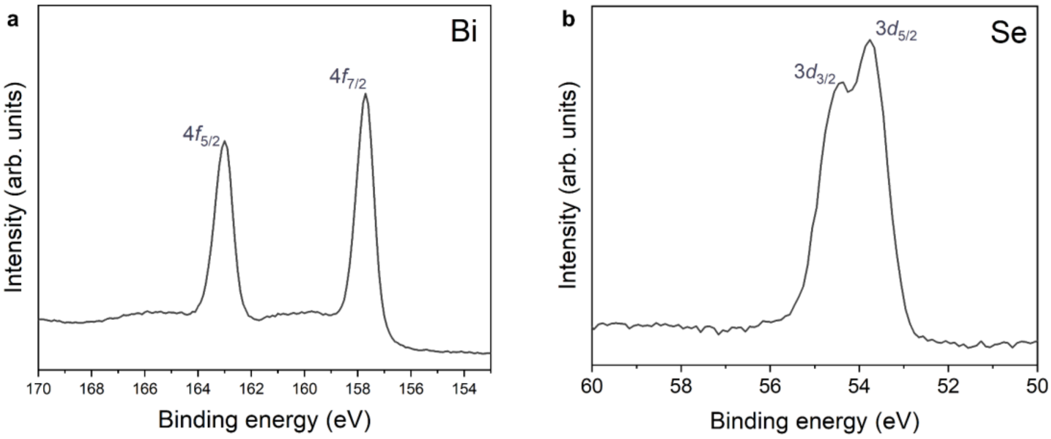

Supplementary Figure 4 | XPS characterizations. **a**, XPS spectra of Bi 4*f* core levels of Bi_2Se_3. **b**, XPS spectra of Se 3*d* core levels of Bi_2Se_3.

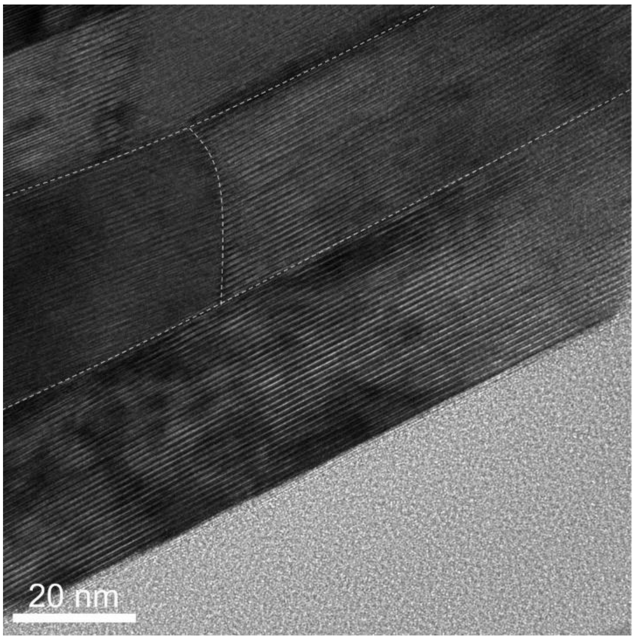

Supplementary Figure 5 | HRTEM image of the film at the boundary area. Dashed lines indicate grain boundaries.

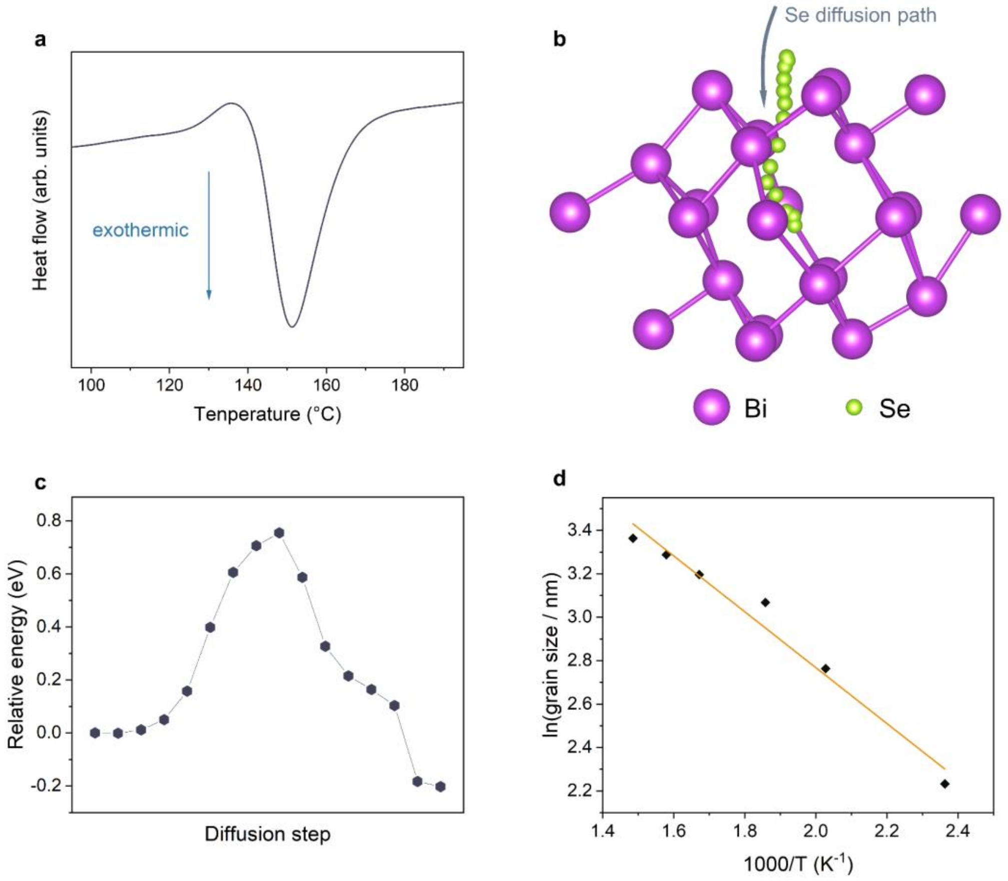

Supplementary Figure 6 | Self-propagating combustion process of the Bi_2Se_3 film. a, DSC curve of the self-propagating combustion process. **b**, Atomic structure showing one Se atom diffusing into the Bi matrix. The grey arrow denotes the motion of the Se atom, and its positions in all diffusion steps are superimposed. **c**, Energy profile as a function of the step along the Se diffusion pathway. **d**, Arrhenius plot of change in crystallite size as a function of PIS temperatures.

Supplementary Note 2

A criterion for the self-propagating combustion process is that the adiabatic temperature (T_{ad}) should be higher than the lower melting point component ($T_{m,L}$)[5], i.e., $T_{ad}/T_{m,L} > 1$. The material fulfills this criterion, but the ignition temperature is lower, and the reaction is faster. In this regard, we propose that the kinetic process (i.e., the thermal diffusion of elements) is responsible for the self-sustained chemical reaction. By considering the one-dimensional semi-finite model, we approximate the Se concentration profile when diffusing into the Bi matrix:

$$C=C_0\left[1\text{-erf}\left(\frac{x}{2\sqrt{Dt}}\right)\right] \quad (1k)$$

where C_0 is the concentration of the Se layer, D is the diffusion coefficient of Se, t is the diffusion time, and erf stands for the error function defined by the indefinite integral:

$$\text{erf}(x)=\frac{2}{\sqrt{\pi}}\int_0^x \exp(-y^2)\,\mathrm{d}y \quad (2k)$$

When significant diffusion occurs (i.e., the concentration of Se at the depth is half of that at the Se/Bi interface), the characteristic diffusion distance (l) can be estimated as[6]

$$l=\sqrt{Dt} \quad (3k)$$

Since the total reaction time is around 1 second, we set the t to 0.1 s. Next, the diffusion coefficient D is simply expressed by an Arrhenius-type relation:

$$D=D_0\exp\left(-\frac{Q}{k_bT}\right) \quad (4k)$$

Where D_0 is the pre-exponential factor, Q is the diffusion activation energy, k_b is the Boltzmann constant, and T is the absolute temperature. From previous literatures[7], we estimate D_0 is in the order of ~ 10^{-2} m^2 s^{-1}. Then, we calculate the theoretical Q using the climbing image nudged elastic band (CI-NEB) method. The diffusion pathway and the energy profile in Supplementary Fig. 6b and c show a diffusion energy barrier of 0.75 eV. Thus, the characteristic diffusion distance at 150 °C is 1.1 μm, which is sufficient to cover the whole thickness of the elemental film. Thus, the self-propagating combustion can be ignited in a solid-state medium (also referred to as solid flame) due to the rapid diffusion between reactants. This phenomenon has been reported in several material systems[8-11].

In the meantime, the formation barrier (ΔG) of Bi_2Se_3 is also investigated. We extract the actual formation energy via the Arrhenius equation and Scherrer equation applied to XRD spectra in Supplementary Fig. 2a[12,13]. The equation is similar to equation (4)[14]:

$$d=A\exp\left(-\frac{\Delta G}{k_bT}\right) \quad (5k)$$

where d is the crystallite size, and A is a constant. Specifically, the crystallite size is determined by measuring the full width at half maximum FWHM of X-ray lines with characteristic (006) peaks. The fitting of the formation barrier ΔG in Supplementary Fig. 6d gives a value of 0.11 eV, which is a very small value and further indicates the low processing temperature of PIS.

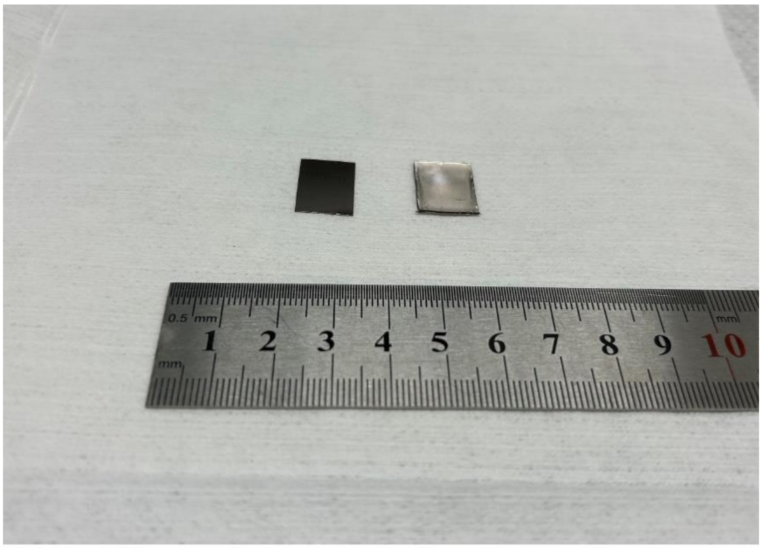

Supplementary Figure 7 | Photograph of PET substrates after PIS (left) and CFA (right).

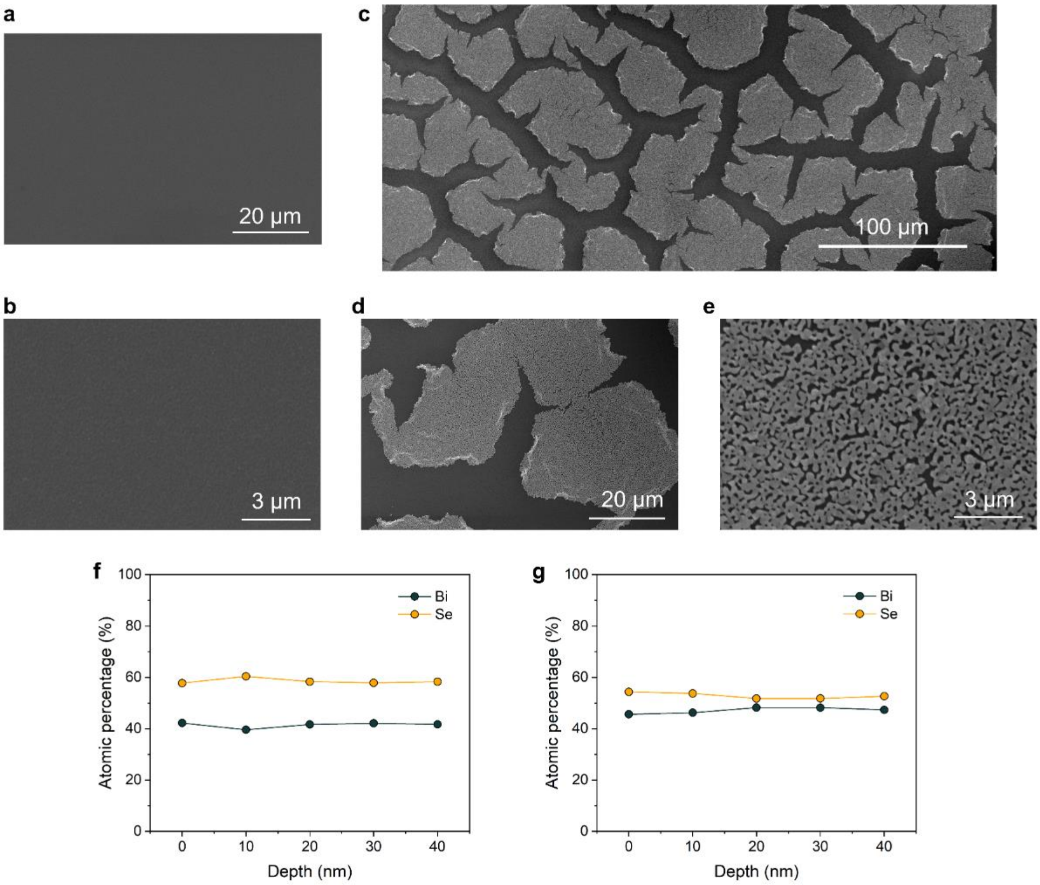

Supplementary Figure 8 | SEM images indicate that prolonged annealing destroys the film quality. a,b, Low-magnification (**a**) and high-resolution (**b**) SEM images of the Bi_2Se_3 film after PIS. **c-e**, Larger SEM image (**c**) showing the degradation of the Bi_2Se_3 film after CFA and detailed morphologies under low-magnification (**d**) and high-resolution (**e**). **f,g**, XPS depth profile analyses of the Bi_2Se_3 film after PIS (**f**) and CFA (**g**)

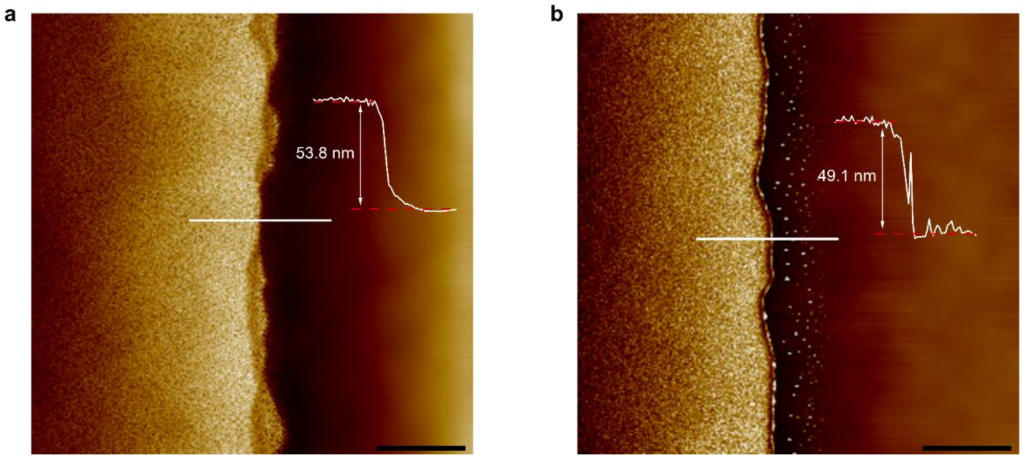

Supplementary Figure 9 | AFM images of the terraced Bi_2Se_3 film before (a) and after (b) PIS. All scale bars are 10 µm.

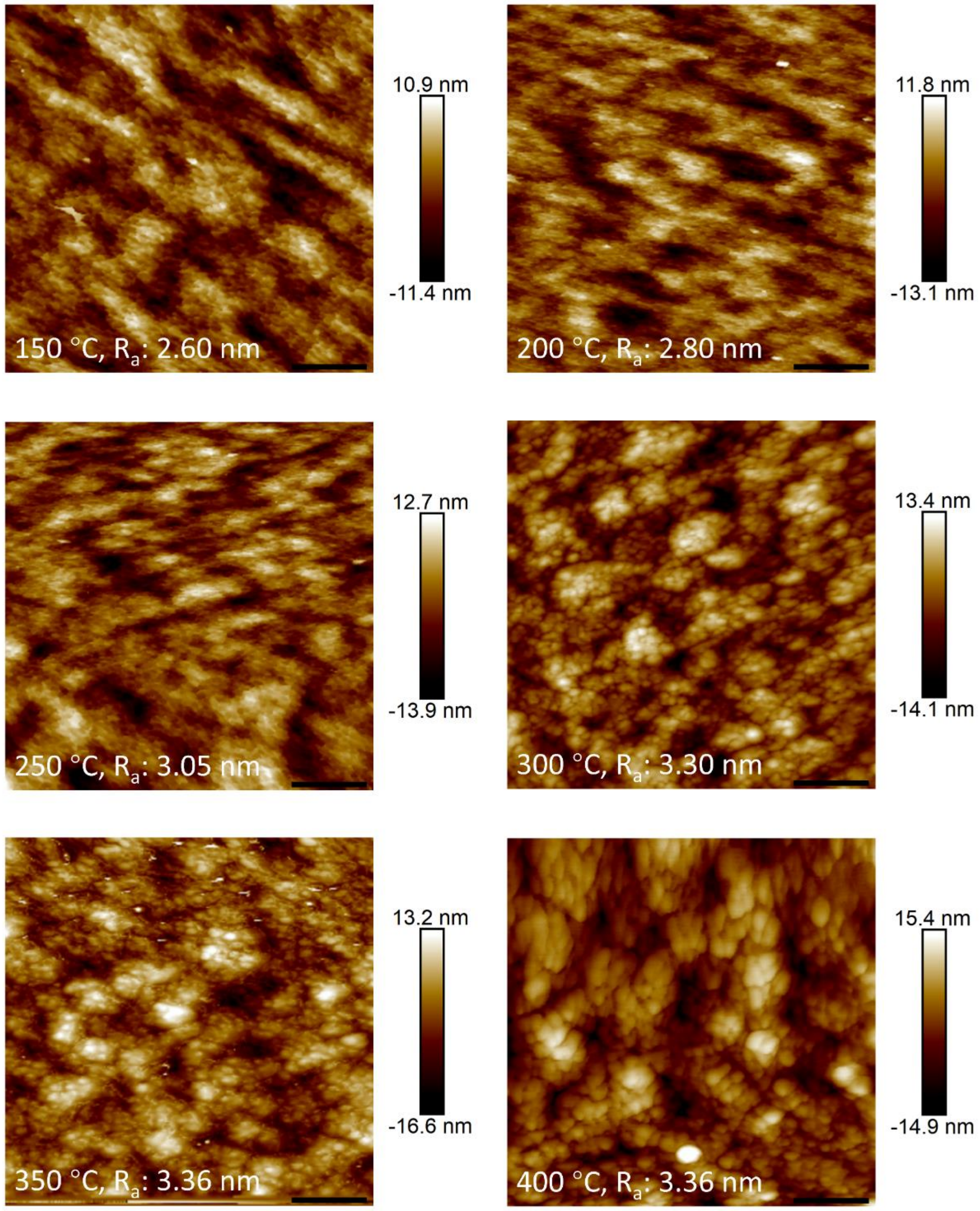

Supplementary Figure 10 | AFM images of the Bi_2Se_3-based films after different PIS temperatures. All scale bars are 200 nm.

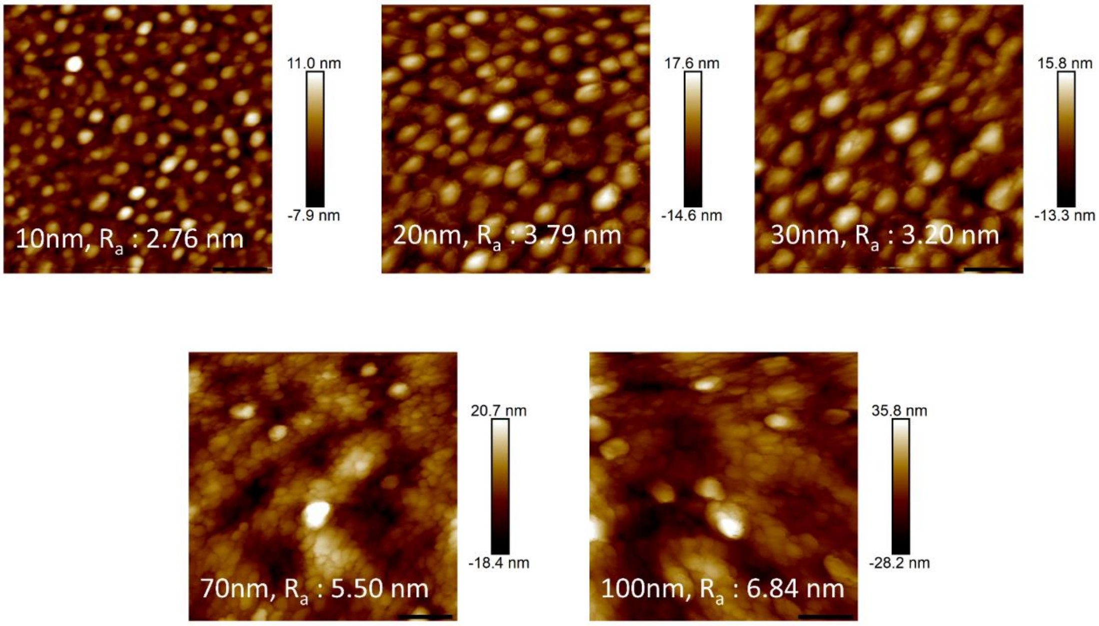

Supplementary Figure 11 | AFM images of the Bi_2Se_3 films with different thicknesses after 300 °C PIS treatment. All scale bars are 200 nm.

Supplementary Note 3

The temperature profile is derived from the law of conservation of energy to a small element of volume, which is extended from classical thermodynamics. This gives rise to the equation of change for internal energy:

$$\frac{\partial}{\partial t}(\rho U)+\nabla\cdot(\rho U\mathbf{v})=-\nabla\cdot\mathbf{q}-p(\nabla\cdot\mathbf{v})+\boldsymbol{\tau}:\nabla\mathbf{v} \tag{6k}$$

Here, t is time, ρ is the density of the material, U is the internal energy, $\mathbf{q}$ is the vector of heat conduction, $\mathbf{v}$ is the velocity vector, p is the pressure, and $\boldsymbol{\tau}$ is the viscous momentum-flux tensor. The first term and the second term on the left side of the equation (6) refer to the rate of increase in internal energy per unit volume and the net rate of addition of internal energy by convective transport, respectively. While terms on the right side refer to the rate of internal energy addition by heat conduction per unit volume, the reversible rate of internal energy increase by compression, and the irreversible rate of internal energy increase by viscous dissipation, respectively. We are more interested in enthalpy rather than internal energy. With the following relation:

$$U=H-pV=H-\frac{p}{\rho} \tag{7k}$$

Equation (6) can be rewritten with the assumption that the thermodynamic formula of equation (7) under equilibrium conditions may be applied locally for non-equilibrium systems. We substitute this formula into equation (6) and get:

$$\frac{\partial}{\partial t}(\rho H)+\nabla\cdot(\rho\mathbf{v}H)=-\nabla\cdot\mathbf{q}+\mathbf{v}\cdot\nabla p+\boldsymbol{\tau}:\nabla\mathbf{v}+\frac{\partial p}{\partial t} \tag{8k}$$

The left side of equation (8) can be expanded as:

$$\frac{\partial}{\partial t}(\rho H)+\nabla\cdot(\rho\mathbf{v}H)=H\left[\frac{\partial\rho}{\partial t}+\nabla\cdot(\rho\mathbf{v})\right]+\rho\frac{\partial H}{\partial t}+\rho\mathbf{v}\cdot\nabla H \tag{9k}$$

According to the equation of continuity, the first term on the right side of equation (9) is zero. Thus, equation (8) can be rewritten as:

$$\rho\frac{\partial H}{\partial t}+\rho\mathbf{v}\cdot\nabla H=-\nabla\cdot\mathbf{q}+\mathbf{v}\cdot\nabla p+\boldsymbol{\tau}:\nabla\mathbf{v}+\frac{\partial p}{\partial t} \tag{10k}$$

Now, we can use temperature to get the expression for the above enthalpy equation. The enthalpy is described with temperature and pressure by the following differential relation:

$$\mathrm{d}H=C_{\mathrm{p}}\mathrm{d}T+\frac{1}{\rho}\left[1+\left(\frac{\partial\ln\rho}{\partial\ln T}\right)_p\right]\mathrm{d}p \tag{11k}$$

where C_{p} and V is the heat compacity under constant pressure and the unit volume, respectively. Then, we can derive the equation of change for temperature by substituting equation (11) into equation (10):

$$\rho C_{\mathrm{p}}\mathrm{d}T=-\nabla\cdot\mathbf{q}+\boldsymbol{\tau}:\nabla\mathbf{v}-\left(\frac{\partial\ln\rho}{\partial\ln T}\right)_p\left(\frac{\partial p}{\partial t}+\mathbf{v}\cdot\nabla p\right) \tag{12k}$$

The heat conduction is described by Fourier's law with:

$$\mathbf{q}=-k\nabla T \tag{13k}$$

where, k is the thermal conductivity. In this work, the substrate is locally heated by laser illumination in an ambient environment. Hence, the pressure is constant, and the viscous dissipation is omitted. We thus have the following form for heat conduction in solids:

$$\rho C_{\mathrm{p}}\frac{\partial T}{\partial t}=k\nabla^2 T \tag{14k}$$

Thus, we can define the thermal diffusivity α as:

$$\alpha=\frac{k}{\rho C_{\mathrm{p}}} \tag{15k}$$

This parameter quantifies the rate that the temperature smooths out in the material. In a material with high thermal diffusivity, heat moves rapidly because of the fast heat conduction compared to its volumetric heat capacity. This results in a lower temperature gradient on the surface. From the results in Fig. 3g, using a substrate with low heat diffusion is highly efficient in obtaining a larger temperature gradient in the device's channel on the film.

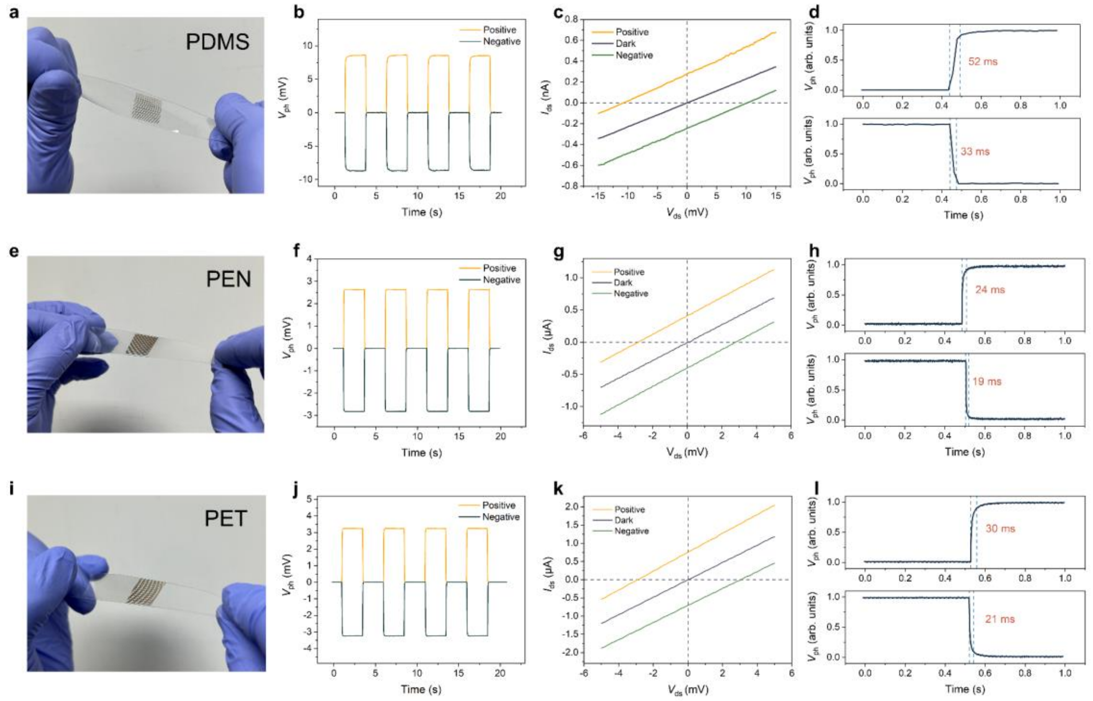

Supplementary Figure 12 | Demonstration of the generality of PIS. a,e,i, Photographs of the Bi_2Se_3 film on PDMS (**a**), PEN (**e**), and PET (**i**) substrates, respectively. **b,f,j**, Photoresponse curves of the Bi_2Se_3 film on PDMS (**b**), PEN (**f**), and PET (**j**) substrates, respectively. **c,g,k**, Output curves of the Bi_2Se_3 film on PDMS (**c**), PEN (**g**), and PET (**k**) substrates, respectively. **d,h,l**, Response times of the Bi_2Se_3 film on PDMS (**d**), PEN (**h**), and PET (**l**) substrates, respectively.

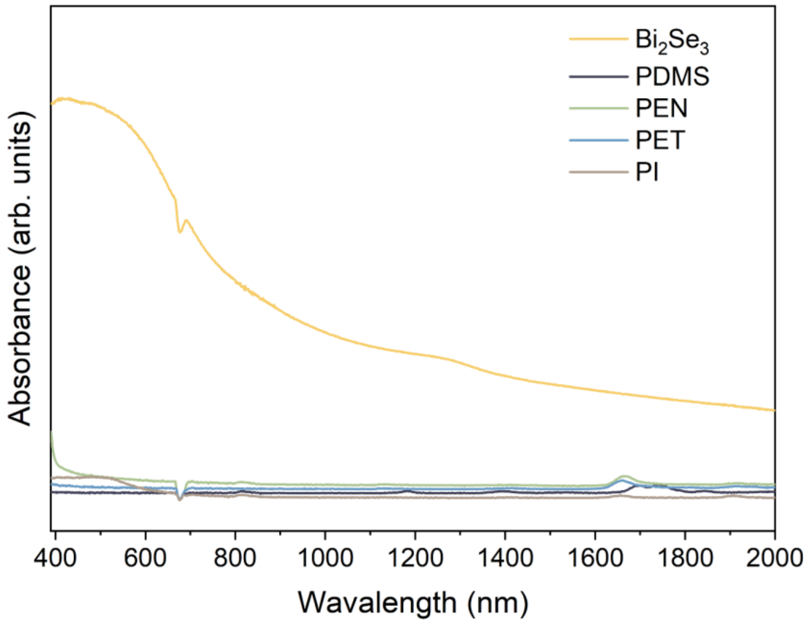

Supplementary Figure 13 | Optical absorption spectra of the PIS Bi_2Se_3 film and four substrates utilized in this work.

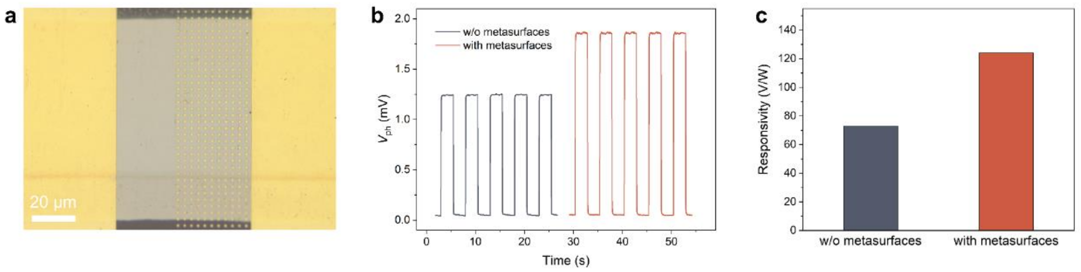

Supplementary Figure 14 | Performance improvement of PTE detectors through meta-material integration. a, Optical image of the Bi_2Se_3 film photodetector integrated with a gold microstructure array. **b**, Temporal response curves with and without integration with meta-materials. **c**, Performance improvement with and without meta-materials.

Supplementary Table 1 | Diffusivity of four typical substrates used in this work.

**Substrate**	**Thermal diffusivity (mm^2 s^{-1})**	**Ref.**
PI	0.21	15
PEN	0.15	16
PET	0.16	16
PDMS	0.07	17

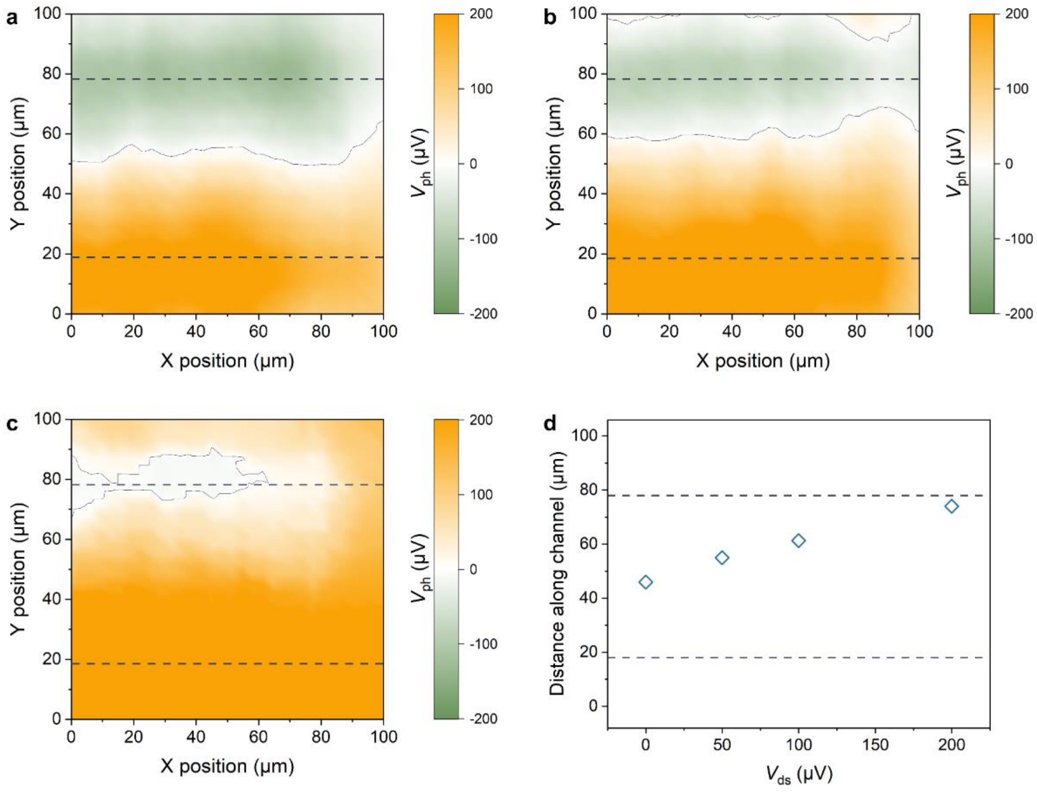

Supplementary Figure 15 | SPVM determined from external biases. a,b,c, SPVM images for the V_{ds} at 50 μV (**a**), 100 μV (**b**), and 200 μV (**c**), respectively. **d**, Zero points along the channel with and without external bias. Grey dashed lines refer to the channel-electrode interface.

Supplementary Note 4

In terms of photodetection mechanisms, it can be classified into several types, and each has a distinct feature that behaves differently in the photo-signal generation. For photodetectors based on the photovoltaic (PV) effect, the photocurrent stems from the separation of electron-hole pairs under the built-in electric field, meaning that the photocurrent generation is localized on the Schottky contact or heterojunction interface[18]. However, our devices have a symmetric structure with no heterojunctions. The output I-V curves with a series of thicknesses show a linear relation, either in the dark or under illumination, which is unattainable for PV detectors due to the asymmetric structure. Therefore, the contribution of the PV effect without external bias is excluded. The response from the photo-Dember (PD) effect only happens at the material-electrode overlapping area where the electron-hole symmetry is broken[19]. Obviously, the photoresponse not only occurs at the electrode region, but also extends well to the channel region with a reversal symmetry, which can rule out the effect of the PD effect. Overall, both PV and PD effects cannot explain our observations, and only the PTE effect has a linear output and nonlocal photoresponse. Here, the Seebeck coefficient of Bi_2Se_3 within the channel is assumed to be a constant due to the uniformity of the film. In this regard, a large temperature gradient across the device via local heat is of great importance for PTE detectors.

Supplementary Table 2 | Comparison of the Seebeck coefficients of Bi_2Se_3 films synthesized by conventional methods.

**Method**	**Seebeck coefficient (μV K^{-1})**	**Year**	**Ref.**
Hydrothermal method	-78	2010	20
Hydrothermal method	-113	2010	21
CVD	-99.9	2021	22
Molecular beam epitaxy	-102.8	2022	23
Magnetron sputtering	-153	2022	24
PIS	-111	2023	This work

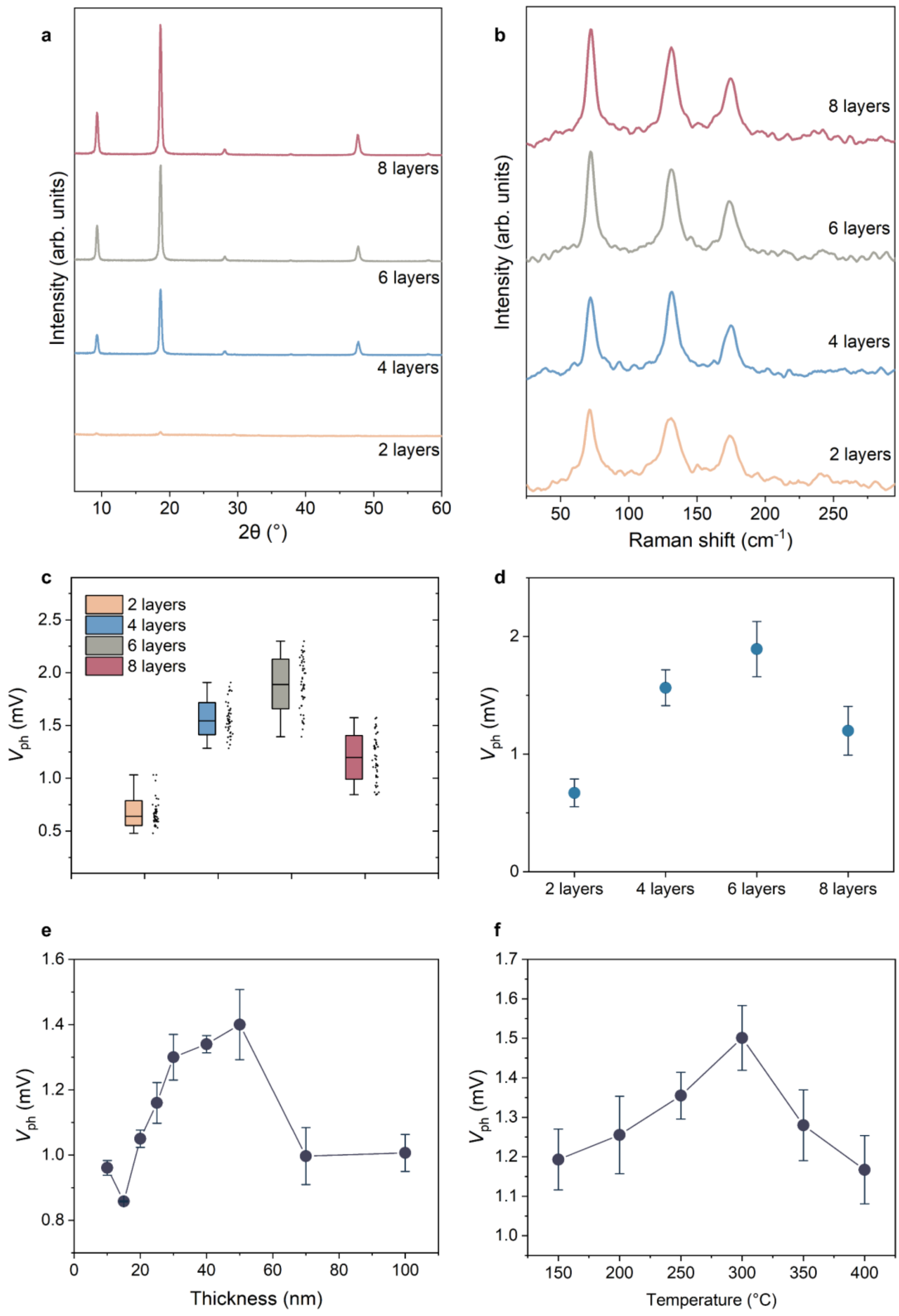

Supplementary Figure 16 | Layer-dependent performance of Bi_2Se_3 films. a,b, XRD (**a**) and Raman (**b**) spectra of Bi_2Se_3 films with various elemental stacking layers after PIS. **c,d**, Photovoltage of Bi_2Se_3 films with various elemental stacking layers after PIS. **e,f**, Photovoltage of Bi_2Se_3 films with different thicknesses. All error bars indicate standard deviations of photovoltages obtained from 50 individual devices.

Supplementary Note 5

To further improve the performance of the PTE film, we probe the performance of PTE films by tuning the thickness and the number of stacking layers. The XRD and Raman spectra are shown in Supplementary Fig. 16a and b. Note that alternative stacking above 4 layers has conspicuous (00*l*) peaks, while the crystallinity for only 2 layers is inferior. Since 2-layer stacking has a thicker elemental layer, it results in a long-range interdiffusion and impedes grain growth. With more stacking layers, the photovoltage ascends first and descends for 8-layer stacking. As the thickness is fixed to 50 nm, excessive layers lead to a thinner individual layer. Since each layer is deposited via thermal evaporation, if the deposited film is too thin, it will exhibit an island-like morphology (typically less than 6 nm), which means that the film becomes discontinuous and leads to the poor quality of Bi_2Se_3 film. As a result, the performance of the PTE degrades (Supplementary Fig. 16c and d). Similar reasons can also account for thickness-dependent photovoltage (Supplementary Fig. 16e). The photoresponse also varies with different PIS processes. Under increasing PIS temperature, the maximum value is found at 300 °C (Supplementary Fig. 16f), which is ascribed to the improved crystallinity below 300 °C and the possible Se sublimation that possibly destroys the integrity of the film at high temperature (above 300 °C).

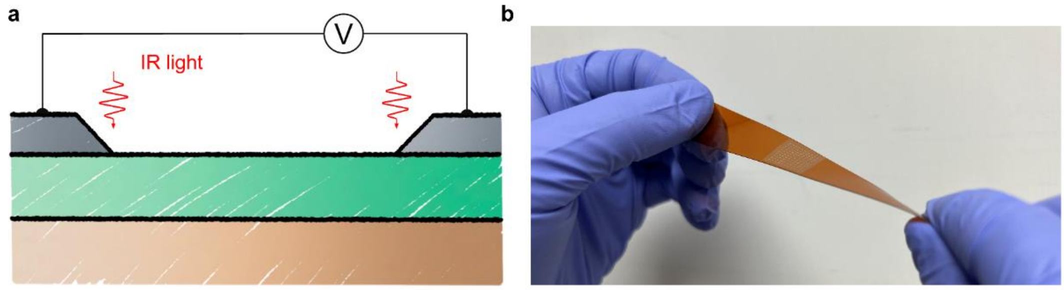

Supplementary Figure 17 | Artist's rendition of the Bi_2Se_3 detector under local IR illumination on either side of the channel (a) and photograph of Bi_2Se_3 film on a twisted PI substrate (b).

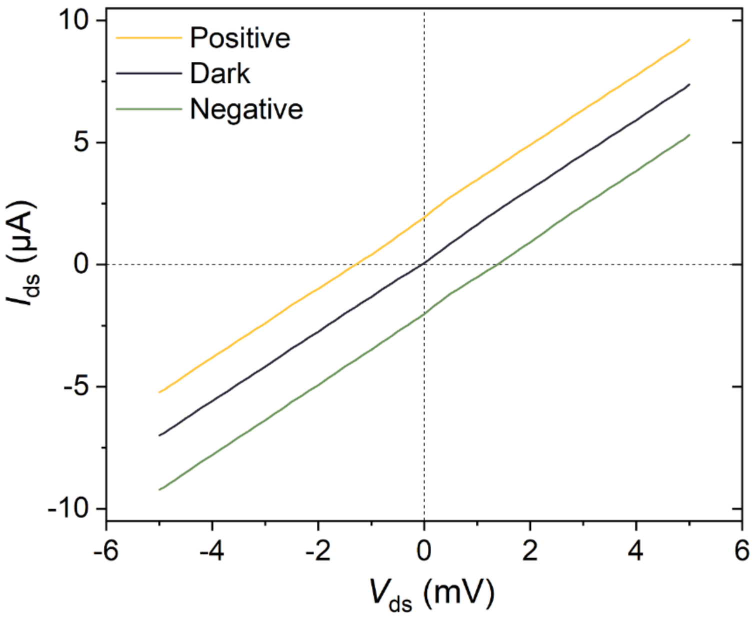

Supplementary Figure 18 | I-V curves in the dark and under illumination.

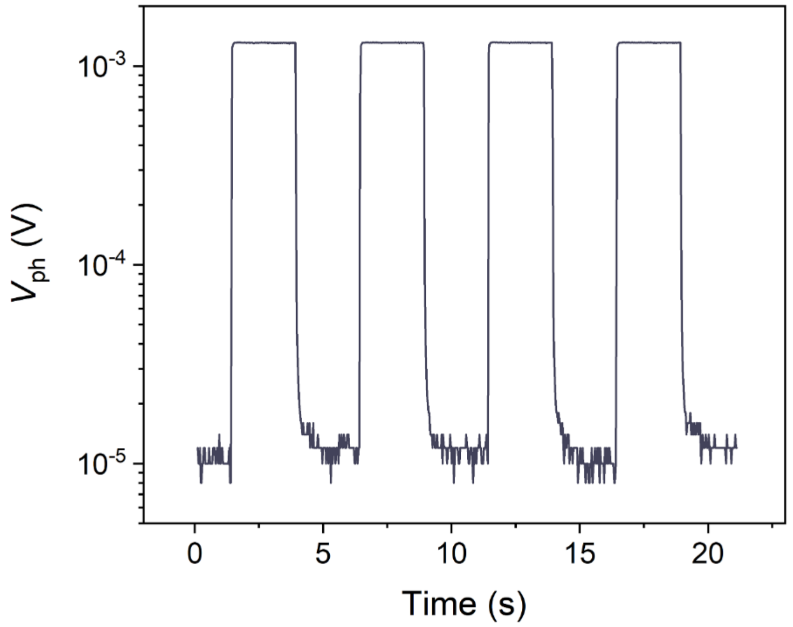

Supplementary Figure 19 | Reproducible on/off switch with a logarithmic *y*-axis.

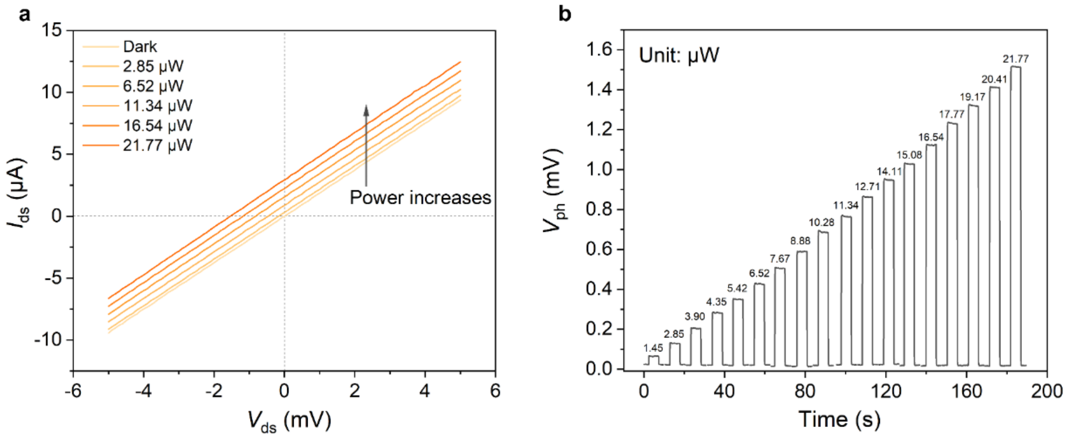

Supplementary Figure 20 | I-V curves under increasing laser powers (a) and temporal response under increasing laser powers (b).

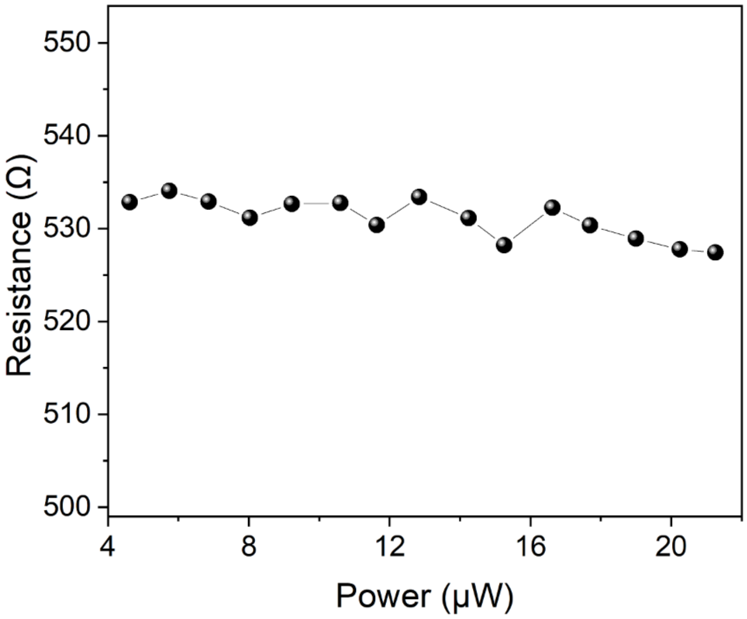

Supplementary Figure 21 | Extracted resistance of the device with a 60 μm channel length under increasing IR illumination power.

Supplementary Table 3 | Detailed comparison of recent PTE photodetectors.

Material	Processing method	Fabrication scalability	Spectral range	Responsivity	Response time	Ref.
Bi_2Te_3/Sb_2Se_3	Magnetron sputtering (350 °C)	High	646 nm	38 V W^{-1}	155 µs	25
MoS_2	Mechanical exfoliation	Low	700 nm	30 V W^{-1}	N.A.	26
Ti-CNT-Pd	Electron beam evaporation	Medium	375-1550 nm	0.68 V W^{-1}	7.23 ms	27
Ag_2Te-Te NWs	Langmuir-Blodgett technique	Low	450 nm	4.1 V W^{-1}	0.48/0.62 s	28
Bi_2Se_3 NWs	CVD	Medium	514 nm	2.6 V W^{-1}	N.A.	29
SWCNT film	CVD	Medium	660 nm	1 V W^{-1}	0.6 s	30
Graphene	Mechanical exfoliation	Low	457-1550 nm	0.52 V W^{-1}	N.A.	31
Ag film/ CNT film	Van der Waal stacking	Low	375-1064 nm	40.4 mV W^{-1}	49/404 ms	32
Black Phosphorus	Mechanical exfoliation	Low	532-1550 nm	5 mA W^{-1}	N.A.	33
Cd_3As_2	CVD (650 °C)	Medium	633 nm	5.88 mA W^{-1}	N.A.	34
Graphene p-n junction	Mechanical exfoliation	Low	850 nm	5 mA W^{-1}	N.A.	35
$MoTe_2$	Mechanical exfoliation	Low	532 nm	0.4 mA W^{-1}	43 µs	36
Bi_2Se_3	PIS (150-300 °C)	High	405-1550 nm	71.9 V W^{-1}	49.7/49.5 ms	This work

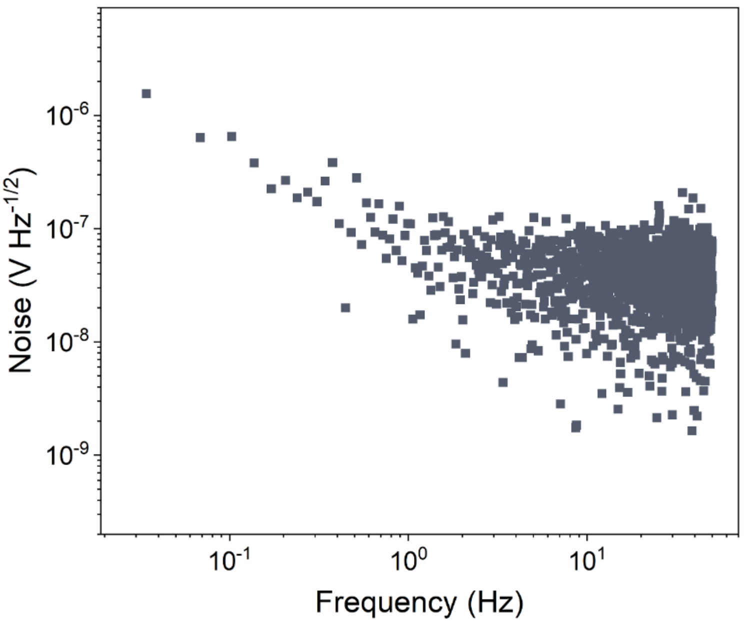

Supplementary Figure 22 | Spectral density of voltage noise under zero bias.

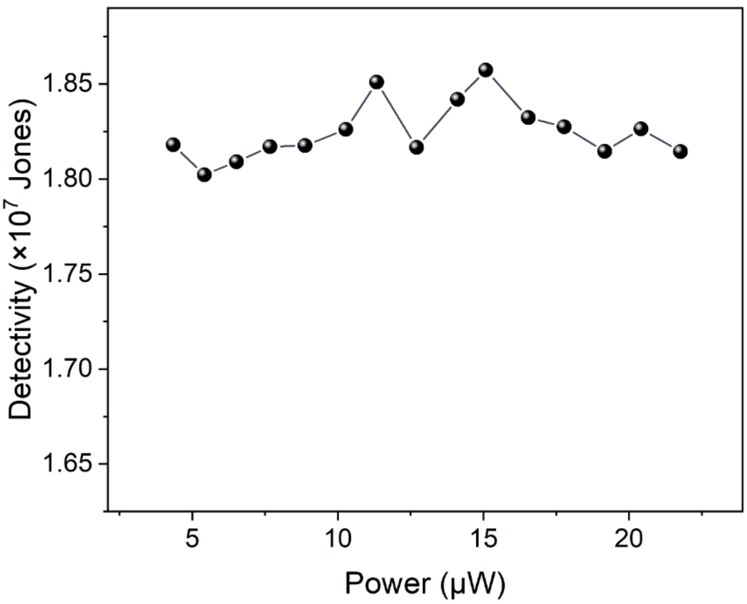

Supplementary Figure 23 | Plot of detectivity *D as a function of incident IR laser power.**

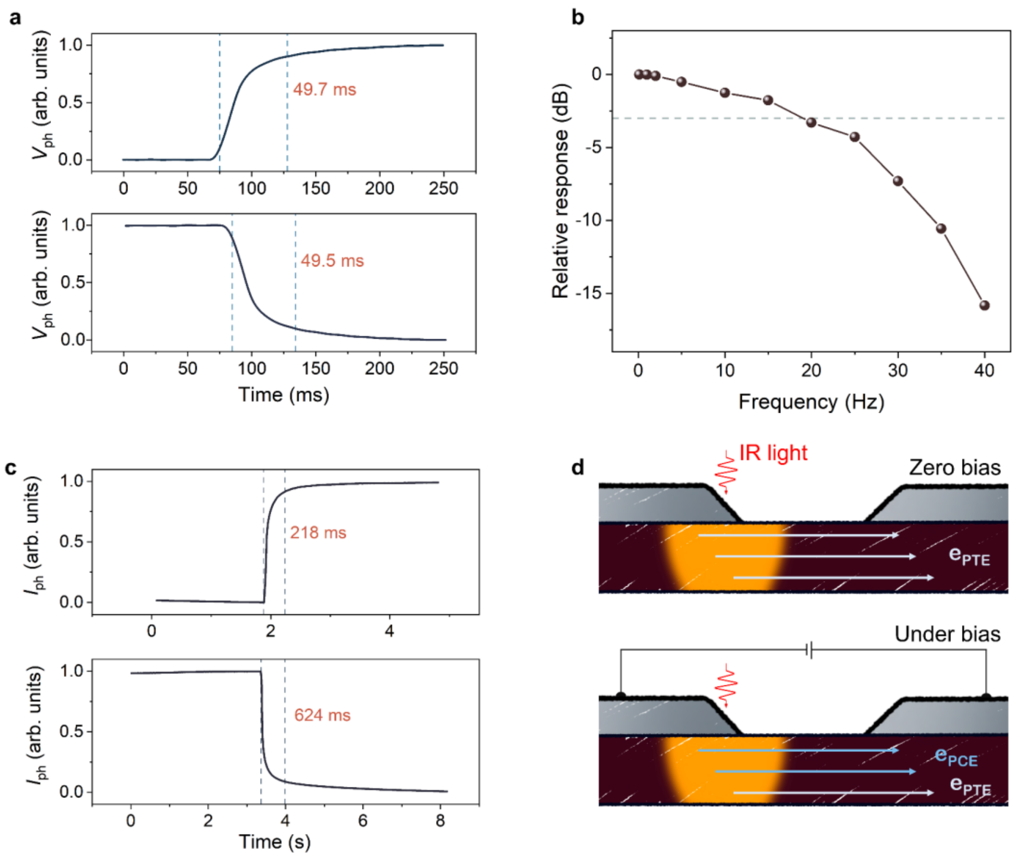

Supplementary Figure 24 | Time-resolved photoresponse of the PTE detector. a, Response time of the device under zero bias. **b**, Frequency response showing a 3 dB frequency cutoff at ~ 20 Hz. **c**, Time-resolved response with an external bias of 1 V when the light spot is positioned at the center of the device channel. **d**, Artist's rendition of the contribution of PTE and photoconductive effect (PCE) under zero bias and 1 V bias.

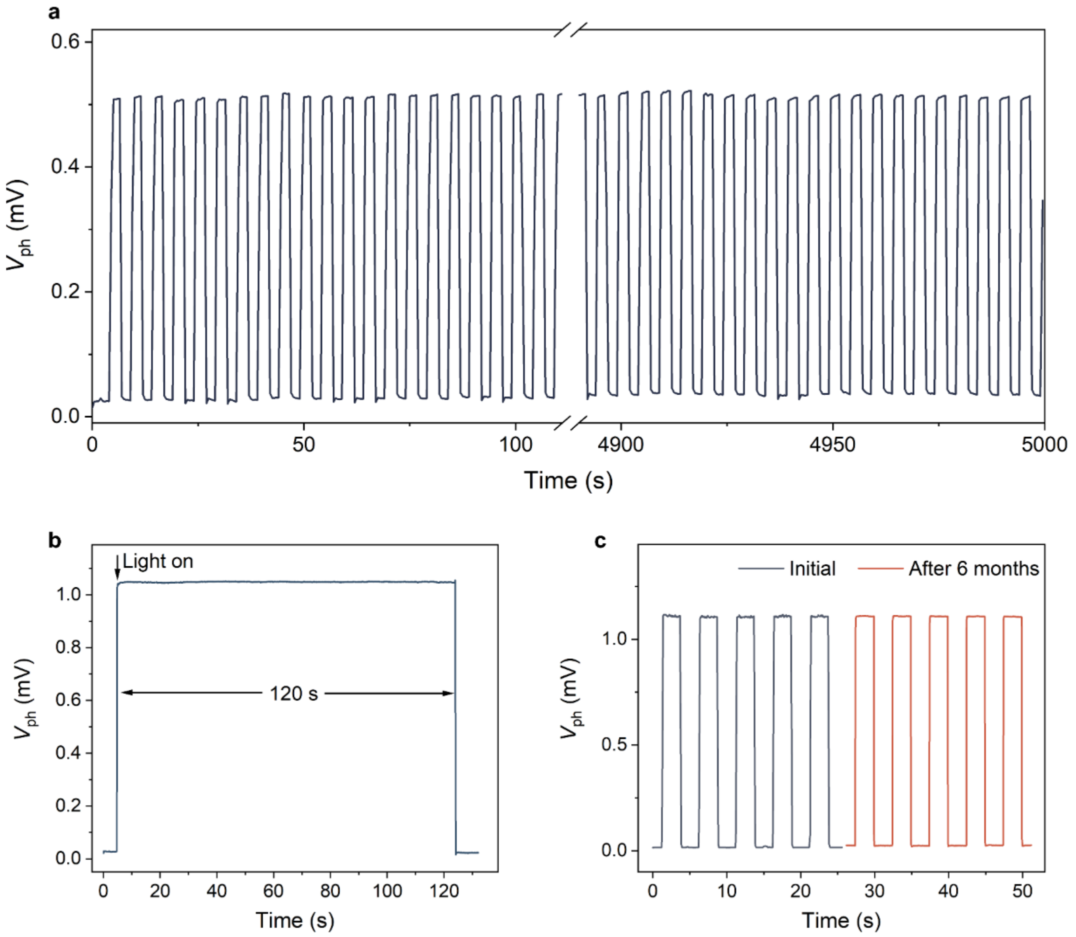

Supplementary Figure 25 | Stability of the Bi_2Se_3 PTE photodetector. a, Response stability as a function of time after 1000 on-off illumination cycles. **b**, Photovoltage stability under continuous irradiation. The device shows a stable photovoltage within 120 s. **c**, Environmental stability of the Bi_2Se_3 detector. The photovoltage of the device is stored in an ambient environment for 6 months. The curve is shifted horizontally for clarity.

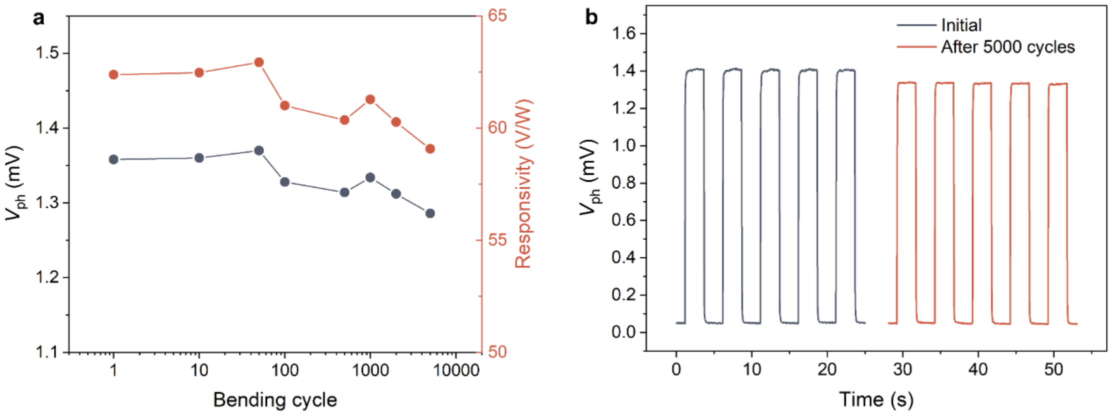

Supplementary Figure 26 | Bending stability of the PTE photodetector. a, Photovoltage and responsivity after different bending cycles. **b**, Photoresponse curves before and after bending test. The bending radius is 5 mm.

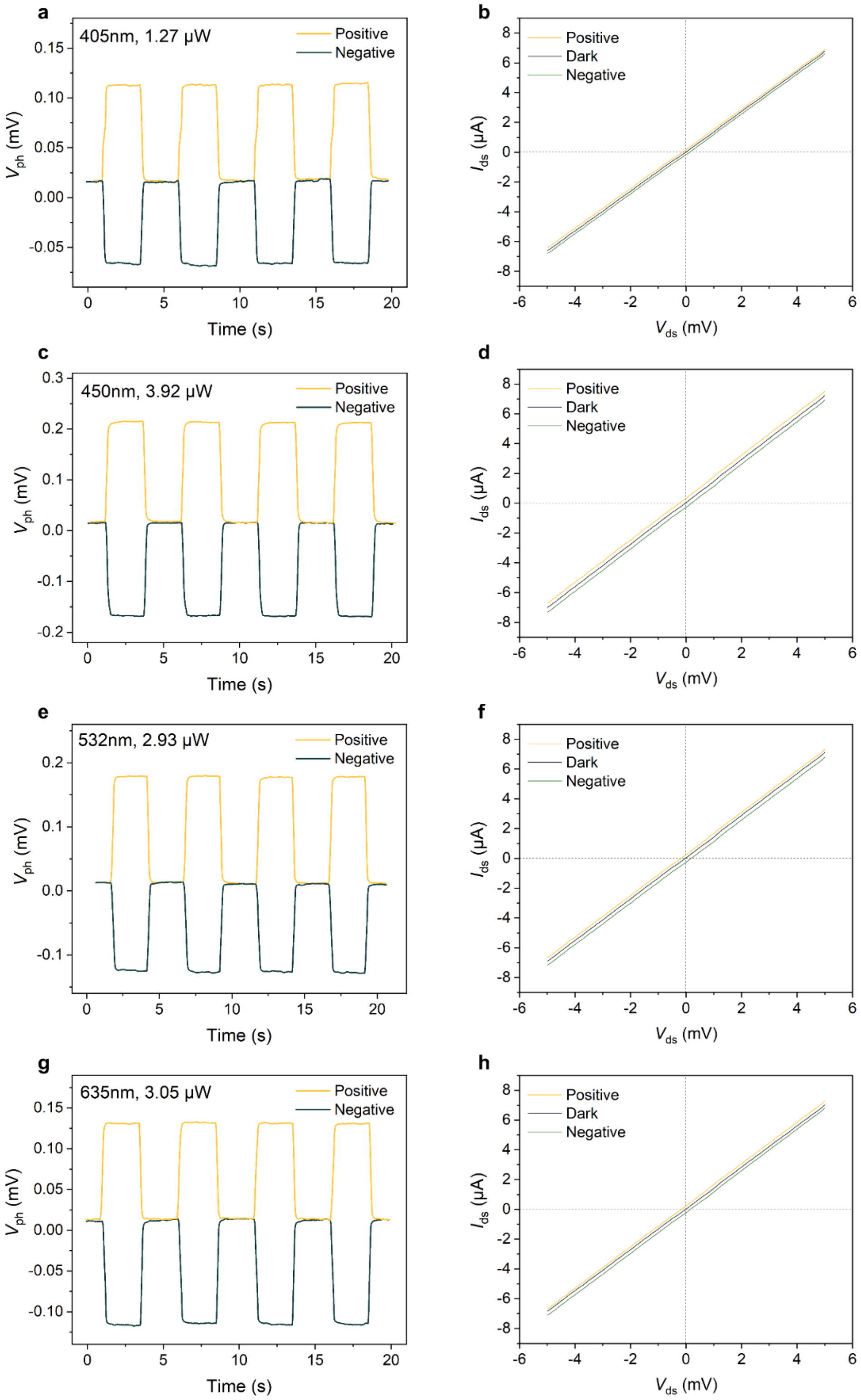

Supplementary Figure 27 | Wide-spectrum response. a,c,e,g, Opposite photoresponses at wavelengths of 405 nm (**a**), 450 nm (**c**), 532 nm (**e**), and 635 nm (**g**), respectively. **b,d,f,h**, Output curves at 405 nm (**b**), 450 nm (**d**), 532 nm (**f**), and 635 nm (**h**), respectively.

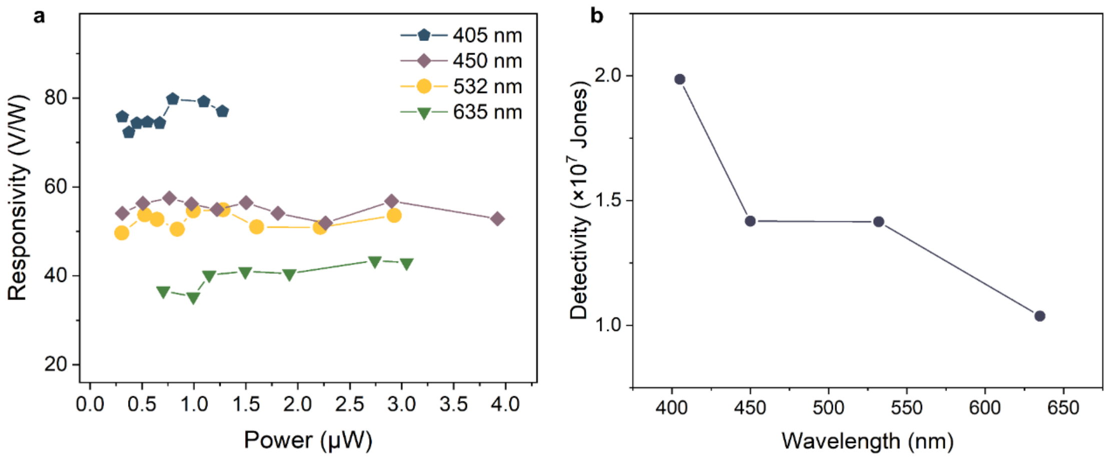

Supplementary Figure 28 | Performance at the visible range. a, Compiled responsivity R as a function of the laser power at the visible range. **b**, Plot of detectivity D^* as a function of incident wavelength.

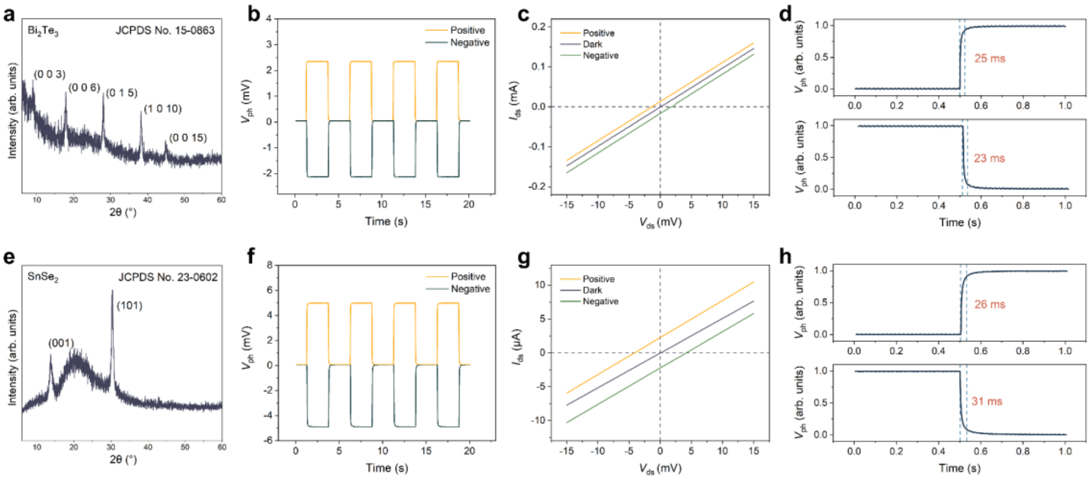

Supplementary Figure 29 | Applicability of PIS on other materials. a,e, XRD spectra suggesting the formation of Bi_2Te_3 and $SnSe_2$ film after low-temperature (150 °C) PIS. **b,f**, Photoresponse curves of Bi_2Te_3 (**b**) and $SnSe_2$ (**f**), respectively. **c,g**, Output curves of Bi_2Te_3 (**c**) and $SnSe_2$ (**g**), respectively. **d,h**, Response times of Bi_2Te_3 (**d**) and $SnSe_2$ (**h**), respectively.

Supplementary References

1. Wang, Y. et al. Near-Infrared Annihilation of Conductive Filaments in Quasiplane $MoSe_2/Bi_2Se_3$ Nanosheets for Mimicking Heterosynaptic Plasticity. *Small* **15,** e1805431 (2019).
2. He, Y. et al. The structure and properties of nanosize crystalline silicon films. *J. Appl. Phys.* **75,** 797-803 (1994).
3. Richter, W. & Becker, C. R. A Raman and far-infrared investigation of phonons in the rhombohedral V2–VI3 compounds Bi_2Te_3, Bi_2Se_3, Sb_2Te_3 and $Bi_2(Te_{1-x}Se_x)_3$ (0<x<1), $(Bi_{1-y}Sb_y)_2Te_3$ (0<y<1). *Phys. Status Solidi B* **84,** 619-628 (1977).
4. Zhang, J. et al. Raman spectroscopy of few-quintuple layer topological insulator Bi_2Se_3 nanoplatelets. *Nano Lett.* **11,** 2407-14 (2011).
5. Su, X. et al. Self-propagating high-temperature synthesis for compound thermoelectrics and new criterion for combustion processing. *Nat. Commun.* **5,** 4908 (2014).
6. Tan, X. et al. New criteria for the applicability of combustion synthesis: The investigation of thermodynamic and kinetic processes for binary Chemical Reactions. *J. Alloys Compd.* **860,** 158465 (2021).
7. Brätter, P. & Gobrecht, H. Self-Diffusion in Selenium. *Phys. Status Solidi B* **37,** 869-878 (1970).
8. Shuck, C. E. et al. Solid-flame: Experimental validation. *Combust. Flame* **163,** 487-493 (2016).
9. Fritz, G. M. et al. Characterizing solid-state ignition of runaway chemical reactions in Ni-Al nanoscale multilayers under uniform heating. *J. Appl. Phys.* **118,** 135101 (2015).
10. Neuhauser, T. et al. The role of two-stage phase formation for the solid-state runaway reaction in Al/Ni reactive multilayers. *Appl. Phys. Lett.* **117,** 011902 (2020).
11. Mukasyan, A. S. et al. The Solid Flame Phenomenon: A Novel Perspective. *Adv. Eng. Mater.* **20,** 1701065 (2018).
12. Ghosh, S., Divya, D., Remani, K. C. & Sreeremya, T. S. Growth of monodisperse nanocrystals of cerium oxide during synthesis and annealing. *J. Nanopart. Res.* **12,** 1905-1911 (2009).
13. Doiphode, R. L., Murty, S. V. S. N., Prabhu, N. & Kashyap, B. P. Grain growth in calibre rolled Mg–3Al–1Zn alloy and its effect on hardness. *J. Magnes. Alloy* **3,** 322-329 (2015).
14. Xu, X. et al. Seeded 2D epitaxy of large-area single-crystal films of the van der Waals semiconductor 2H $MoTe_2$. *Science* **372,** 195-200 (2021).
15. Morikawa, J. & Hashimoto, T. Thermal diffusivity of aromatic polyimide thin films by temperature wave analysis. *J. Appl. Phys.* **105,** 113506 (2009).
16. Morikawa, J. & Hashimoto, T. Study on thermal diffusivity of poly(ethylene terephthalate) and poly(ethylene naphthalate). *Polymer* **38,** 5397-5400 (1997).
17. Stancu, V. et al. Influences of Dispersions' Shapes and Processing in Magnetic Field on Thermal Conductibility of PDMS-Fe_3O_4 Composites. *Materials* **14,** (2021).
18. Lee, E. J. et al. Contact and edge effects in graphene devices. *Nat. Nanotechnol.* **3,** 486-90 (2008).
19. Li, G. et al. Fast Photothermoelectric Response in CVD-Grown $PdSe_2$ Photodetectors with In-Plane Anisotropy. *Adv. Funct. Mater.* **31,** 2104787 (2021).
20. Sun, Z., Liufu, S., Chen, X. & Chen, L. Enhancing thermoelectric performance of bismuth selenide films by constructing a double-layer nanostructure. *CrystEngComm* **12,** 2672 (2010).
21. Sun, Z., Liufu, S. & Chen, L. Synthesis and characterization of nanostructured bismuth selenide thin films. *Dalton Trans.* **39,** 10883-7 (2010).
22. Chen, Q. et al. Morphology Optimization of Bi_2Se_3 Thin Films for Enhanced Thermoelectric Performance. *Cryst. Growth Des.* **21,** 6737-6743 (2021).

23. Kim, D., Yang, C. & Park, Y. D. Towards More Accurate Determination of the Thermoelectric Properties of Bi_2Se_3 Epifilms by Suspension via Nanomachining Techniques. *Sensors (Basel)* **22,** (2022).
24. Gautam, S. et al. Structural, Electronic and Thermoelectric Properties of Bi2Se3 Thin Films Deposited by RF Magnetron Sputtering. *J. Electron. Mater.* **51,** 2500-2509 (2022).
25. Mauser, K. W. et al. Resonant thermoelectric nanophotonics. *Nat. Nanotechnol.* **12,** 770-775 (2017).
26. Buscema, M. et al. Large and tunable photothermoelectric effect in single-layer MoS_2. *Nano Lett.* **13,** 358-63 (2013).
27. Liu, Y. et al. High-Performance Ultrabroadband Photodetector Based on Photothermoelectric Effect. *ACS Appl. Mater. Interfaces* **14,** 29077-29086 (2022).
28. Wang, R. et al. Manipulating Nanowire Structures for an Enhanced Broad-Band Flexible Photothermoelectric Photodetector. *Nano Lett.* **22,** 5929-5935 (2022).
29. Yan, Y. et al. Topological surface state enhanced photothermoelectric effect in Bi_2Se_3 nanoribbons. *Nano Lett.* **14,** 4389-94 (2014).
30. He, X. et al. Photothermoelectric p-n junction photodetector with intrinsic broadband polarimetry based on macroscopic carbon nanotube films. *ACS Nano* **7,** 7271-7 (2013).
31. Echtermeyer, T. J. et al. Photothermoelectric and photoelectric contributions to light detection in metal-graphene-metal photodetectors. *Nano Lett.* **14,** 3733-42 (2014).
32. Lv, B. et al. Local large temperature difference and ultra-wideband photothermoelectric response of the silver nanostructure film/carbon nanotube film heterostructure. *Nat. Commun.* **13,** 1835 (2022).
33. Engel, M., Steiner, M. & Avouris, P. Black phosphorus photodetector for multispectral, high-resolution imaging. *Nano Lett.* **14,** 6414-7 (2014).
34. Wang, Q. et al. Ultrafast Broadband Photodetectors Based on Three-Dimensional Dirac Semimetal Cd_3As_2. *Nano Lett.* **17,** 834-841 (2017).
35. Gabor, N. M. et al. Hot carrier-assisted intrinsic photoresponse in graphene. *Science* **334,** 648-52 (2011).
36. Lai, J. et al. Anisotropic Broadband Photoresponse of Layered Type-II Weyl Semimetal $MoTe_2$. *Adv. Mater.* **30,** e1707152 (2018).
