# Supplementary material for: Pulse irradiation synthesis of metal chalcogenides on flexible substrates for enhanced photothermoelectric performance
Source: Nat Commun. 2024 Jan 25;15:728. doi: 10.1038/s41467-024-44970-4 (PMC10810900; doi:10.1038/s41467-024-44970-4)
Supplement: Supplementary file 3 — Description of Additional Supplementary Files [file 41467_2024_44970_MOESM3_ESM.pdf]

Description of Additional Supplementary Files

File Name: Supplementary Movie 1
Description: MD simulation of the interdiffusion of Bi atoms and Se atoms |The interdiffusion initiates from the interface between Bi and Se, and the fusion almost completes after 18 ps.

File Name: Supplementary Movie 2
Description: MD simulation after the formation of the Bi2Se3 prime cluster | Once the primary cluster is formed, Bi or Se atoms are prone to attach to the edge of the Bi2Se3 cluster, promoting its lateral crystal growth.

File Name: Supplementary Movie 3
Description: Simultaneous recording of the changes in the film surface temperature and the photovoltage | The temperature rise and decay are synchronized to photovoltage. The surface temperature of the film immediately rises about 3 K as long as the 1550 nm laser turns on.
